# Sulfatase 1 (hSulf-1) reverses basic fibroblast growth factor-stimulated signaling and inhibits growth of hepatocellular carcinoma in animal model

**DOI:** 10.18632/oncotarget.2078

**Published:** 2014-06-08

**Authors:** Gaoya Xu, Weidan Ji, Yinghan Su, Yang Xu, Yan Yan, Shuwen Shen, Xiaoya Li, Bin Sun, Haihua Qian, Lei Chen, Xiaohui Fu, Mengchao Wu, Changqing Su

**Affiliations:** ^1^ Department of Molecular Oncology, Eastern Hepatobiliary Surgical Hospital & National Center of Liver Cancer, The Second Military Medical University, Shanghai, China; ^2^ Department of Pathogen Biology, School of Biology & Basic Medical Sciences, Soochow University, Suzhou, China; ^3^ Department of Biology, Xi'an Jiaotong-Liverpool University, Suzhou, China

**Keywords:** human sulfatase 1, cell cycle, apoptosis, AKT/ERK signaling, hepatocellular carcinoma

## Abstract

The human sulfatase 1 (hSulf-1) gene encodes an endosulfatase that functions to inhibit the heparin-binding growth factor signaling, including the basic fibroblast growth factor (bFGF)-mediated pathway, by desulfating the cell surface heparan sulfate proteoglycans (HSPGs). bFGF could stimulate cell cycle progression and inhibit cell apoptosis, this biological effect can be reversed by hSulf-1. However, molecular mechanisms have not been fully reported. In the current study, by reactivation of hSulf-1 expression and function in the hSulf-1-negative hepatocellular carcinoma (HCC) cell lines and HCC xenograft tumors, we found that hSulf-1 blocked the bFGF effect on the promotion of cell cycle and inhibition of apoptosis. The bFGF-stimulated activation of protein kinase B (AKT) and extracellular signal-regulated kinase (ERK) pathways was suppressed by hSulf-1, which led to a decreased expression of the target genes Cyclin D1 and Survivin, then finally induced cell cycle arrest and apoptosis in HCC cells. Our data suggested that hSulf-1 may be a suitable target for cancer therapy.

## INTRODUCTION

Survival of cancer cells depends on the activation of a series of signaling pathways. In most of cancers, various growth factors and their receptors are overexpressed, such as the basic fibroblast growth factor (bFGF) [1-3], epidermal growth factor (EGF) [4], platelet-derived growth factor (PDGF) [5,6], hepatocyte growth factor (HGF) [3,7] and vascular endothelial growth factor (VEGF) [8]. The factors activate the relative signaling pathways, such as the Ras/Raf/mitogen-extracellular activated protein kinase kinase (MEK)/ extracellular signal-regulated kinase (ERK), phosphatidylinositol-3-kinase (PI3K)/phosphatase and tensin homologue deleted on chromosome ten (PTEN)/Akt/mammalian target of rapamycin (mTOR), that are involved in cancer cell survival via interactions with their specific receptors and then maintain the malignant growth of tumors [9]. The sulfation of N-acetylglucosamine residues of heparan sulfate proteoglycans (HSPGs) on cell surface mediates the interaction or binding of growth factors and their receptors [10], which is a critical link for the cascade activation of signal pathways. The human sulfatase 1 (hSulf-1) is a heparin-degrading endosulfatase, which can desulfate HSPGs and block the binding of growth factors and their receptors, and finally inhibit the activation of growth factor signaling pathways [11,12]. The expression of hSulf-1 protein is positive in normal tissue, but negative or weak positive in majority of various human cancers [11]. Re-expression of hSulf-1 in cancer cells can inhibit cancer cell proliferation and xenograft tumor growth [13]. Therefore, the hSulf-1 can be regarded as an inhibitory factor in cancers and a potential target for cancer therapy.

Loss of cell cycle control and inhibition of cell apoptosis lead to uncontrolled cell proliferation and tumor progression in majority of cancers. bFGF is considered to play an important role in stimulating the proliferation of various cancer cells. It can induce cancer cell proliferation by activating or expressing G1-S phase proteins, including E2F-1, cdks and cyclins, through the reactive oxygen species/phosphoinositide 3-kinase (PI3K)/protein kinase B (AKT) pathway in colon cancer [14,15]. bFGF also can sustain CD44(+)/CD24(-) cell proliferation and promote cell progression through G0/G1 to G2/S phase transition [16]. Inhibition of bFGF mitogenic activity with bFGF-binding peptide may reverse the effects of bFGF and lead to suppression of proliferation, cell cycle arrest at G0/G1 phase, reduction of phospho-ERK1/2 levels in the bFGF-stimulated cells of colon cancer [17], leukemia [18] and melanoma [19].

Since hSulf-1 plays a negative regulatory role in bFGF signaling pathways, and bFGF functions to promote cell cycle progression in human cancers as aforementioned, it is supposed that hSulf-1 can regulate cancer cell cycle and control cancer programmed cell death, such as apoptosis. However, there is no enough evidence to clarify the relationship between hSulf-1 function and cell cycle regulation. In this study, bFGF was used to stimulate the cell cycle progression in HCC cell lines, hSulf-1 was re-expressed by adenoviral vector to block the bFGF-mediated signaling pathways, and the regulatory effects and mechanisms of hSulf-1 on HCC cell cycle control were investigated in the in vitro experiments. The antitumor efficacy of hSulf-1 involved in cell cycle control was also validated in HCC cancer xenografts in nude mice. This study gave a new insight into the function of hSulf-1 as a negative regulator of cell cycle, which will help to design a novel strategy of hSulf-1 gene therapy in HCC target treatment.

## RESULTS

### hSulf-1 reverses the effect of bFGF on cell cycle progression and apoptosis in the hSulf-1-negative HCC cell lines

**Figure 1:**
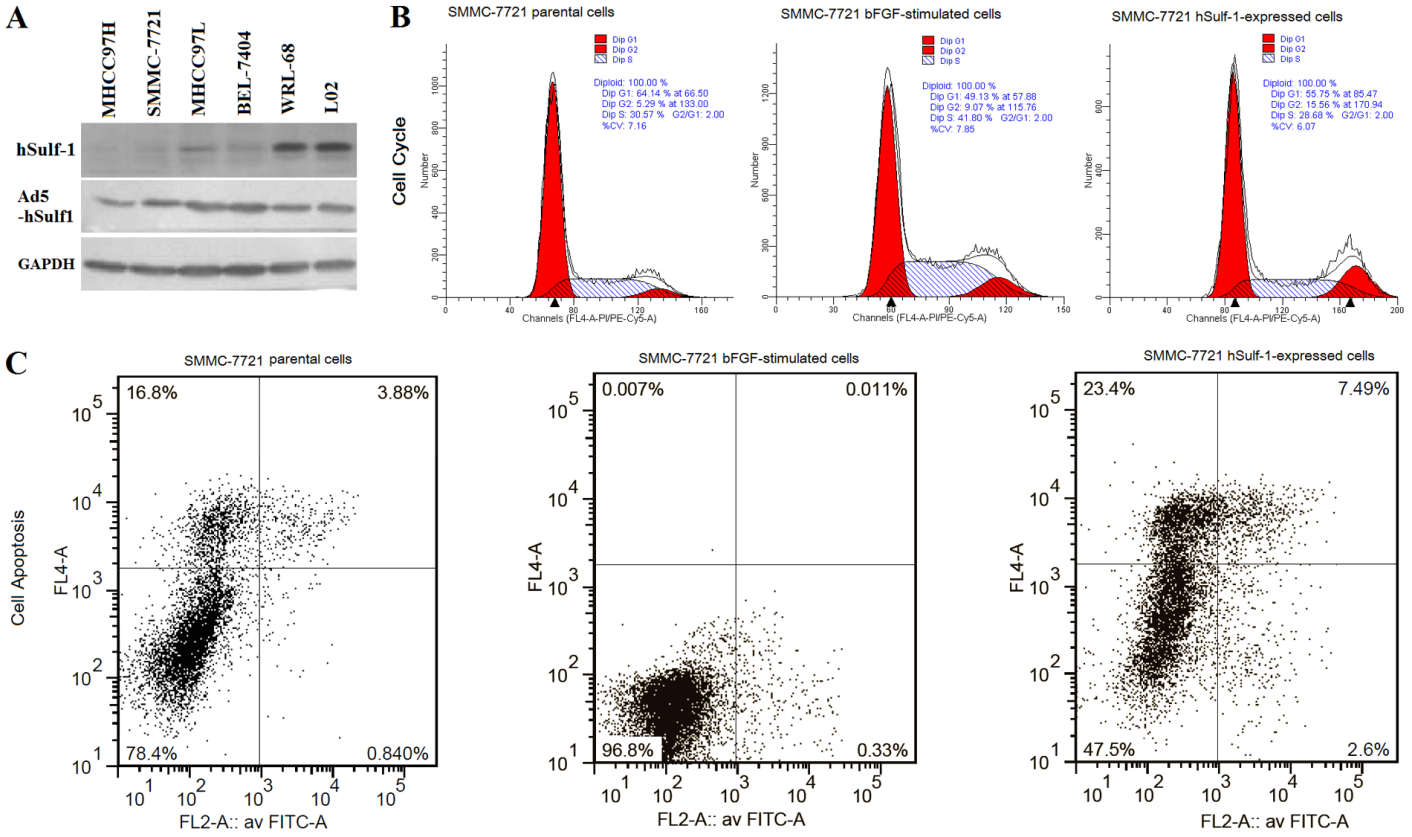
hSulf-1 reactivation reversed the bFGF-stimulated cell cycle progression and apoptosis inhibition in HCC cells (A) SMMC-7721 cells were cultured in 6-well plates at 1×10^6^ cells/well with or without bFGF, and infected with adenovirus Ad5-hSulf1 at a multiplicity of infection (MOI) of 100 pfu/cell. Forty-eight h later, the parental cells, cells cultured in media containing bFGF and cells infected with adenovirus were harvested and the cell lysates were prepared. The expression of hSulf-1 was examined by Western blot. Glyceraldehydes 3-phosphate dehydrogenase (GAPDH) was used as the loading control. (B, C) The harvested cells were stained by propidium iodide (PI) and Annexin V/PI, cell cycle (B) and apoptosis (C) were examined by flow cytometry. The data of all cell lines were showed and analyzed in Table 1.

The HCC and hepatocyte lines were cultured in media containing bFGF. By Western blot, the expression of hSulf-1 was positive in the normal hepatocyte WRL-68 and L02 cell lines, weak positive in the MHCC97L and BEL-7404 cell lines, and completely negative in the MHCC97H and SMMC-7721 cell lines (Fig. 1A, hSulf-1). The cell cycle and cell apoptosis were measured by PI-staining and Annexin V/PI-staining flow cytometry (FCM), respectively, at 48 h later. Compared with the parental cells without bFGF culture, the percentages of G0/G1-phase were decreased and the percentages of S-phase were increased (Fig. 1B), correspondingly, the percentages of cell apoptosis were decreased (Fig. 1C), in the 4 bFGF-stimulated HCC cell lines, particularly in hSulf-1-negative MHCC97H and SMMC-7721 cell lines. Whereas, the changes in cell cycle and apoptosis had no significant difference in the WRL-68, L02, MHCC97L and BEL-7404 cell lines (Table 1).

**Table 1:** Effect of bFGF or/and hSulf-1 on cell cycle and apoptosis in HCC cell lines

Cell Group	G0/G1(%)	S(%)	G2/M(%)	Apoptosis(%)
MHCC97H				
Praental cells	55.83±6.76	40.11±3.45	3.04±1.03	5.40±1.92
bFGF-stimulated cells	45.42±4.32**	52.03±4.23***	2.51±0.66	2.76±0.27*
hSulf-1-expressed cells	53.36±3.23^###^	34.43±2.55^###^	11.92±1.11^###^	8.51±1.58^###^
MHCC97L				
Praental cells	65.02±6.12	26.77±2.45	8.25±1.24	3.71±0.87
bFGF-stimulated cells	62.66±5.67	30.38±2.43*	7.10±2.23	2.22±0.36
hSulf-1-expressed cells	67.82±4.65	22.41±4.86^#^	9.73±2.64	5.35±0.94^#^
SMMC-7721				
Praental cells	64.01±4.01	31.76±2.34	6.23±2.29	4.51±0.87
bFGF-stimulated cells	50.62±2.54***	42.39±4.65***	7.06±1.22	0.32±0.15**
hSulf-1-expressed cells	56.18±2.61^##^	28.42±3.54^##^	15.39±3.34^##^	10.45±2.34^###^
BEL-7404				
Praental cells	61.05±3.54	33.76±4.37	5.28±1.02	2.67±0.46
bFGF-stimulated cells	56.66±6.65	39.38±4.87*	4.08±1.04	1.84±0.27
hSulf-1-expressed cells	59.84±5.43	34.41±2.38	6.99±1.45	3.75±1.24^#^
WRL-68				
Praental cells	72.01±3.52	19.76±4.25	8.23±2.65	2.92±0.32
bFGF-stimulated cells	79.62±6.22	21.38±4.86	9.06±1.86	1.32±0.14
hSulf-1-expressed cells	70.80±5.06	20.41±5.92	8.79±3.65	3.35±1.08^#^
L02				
Praental cells	71.01±4.67	23.24±2.23	5.75±1.68	1.12±0.21
bFGF-stimulated cells	69.03±7.43	24.08±4.34	6.89±2.54	0.98±0.25
hSulf-1-expressed cells	71.99±3.42	21.58±3.62	7.43±0.89	1.27±0.62

Notes: SNK-q test, *P<0.05, **P<0.01, ***P<0.001 compared with the parental cells; ^#^*P*<0.05, ^##^*P*<0.01, ^###^*P*<0.001 compared with the hSulf-1-expressed cells.

Cells cultured in media containing bFGF were infected with adenovirus Ad5-hSulf1. The expression of hSulf-1 was increasingly positive in the 4 HCC cell lines and did not changed in the normal hepatocyte lines (Fig. 1A, Ad5-hSulf1). Correspondingly, compared with the cells cultured in media containing bFGF, the percentages of G0/G1-phase and G2-phase were increased, the percentages of S-phase were decreased, and the percentages of apoptosis were increased in the Ad5-hSulf1-infected HCC cells (Fig. 1B-C). This tendency was also observed in the normal hepatocyte lines but there were no statistically differences between cells infected with and without adenovirus Ad5-hSulf1 (Table 1). The negative control adenovirus Ad5-EGFP did not show any changes on cell cycle and apoptosis.

### hSulf-1 inhibits the activity of bFGF-induced AKT and ERK signaling in HCC cells

**Figure 2:**
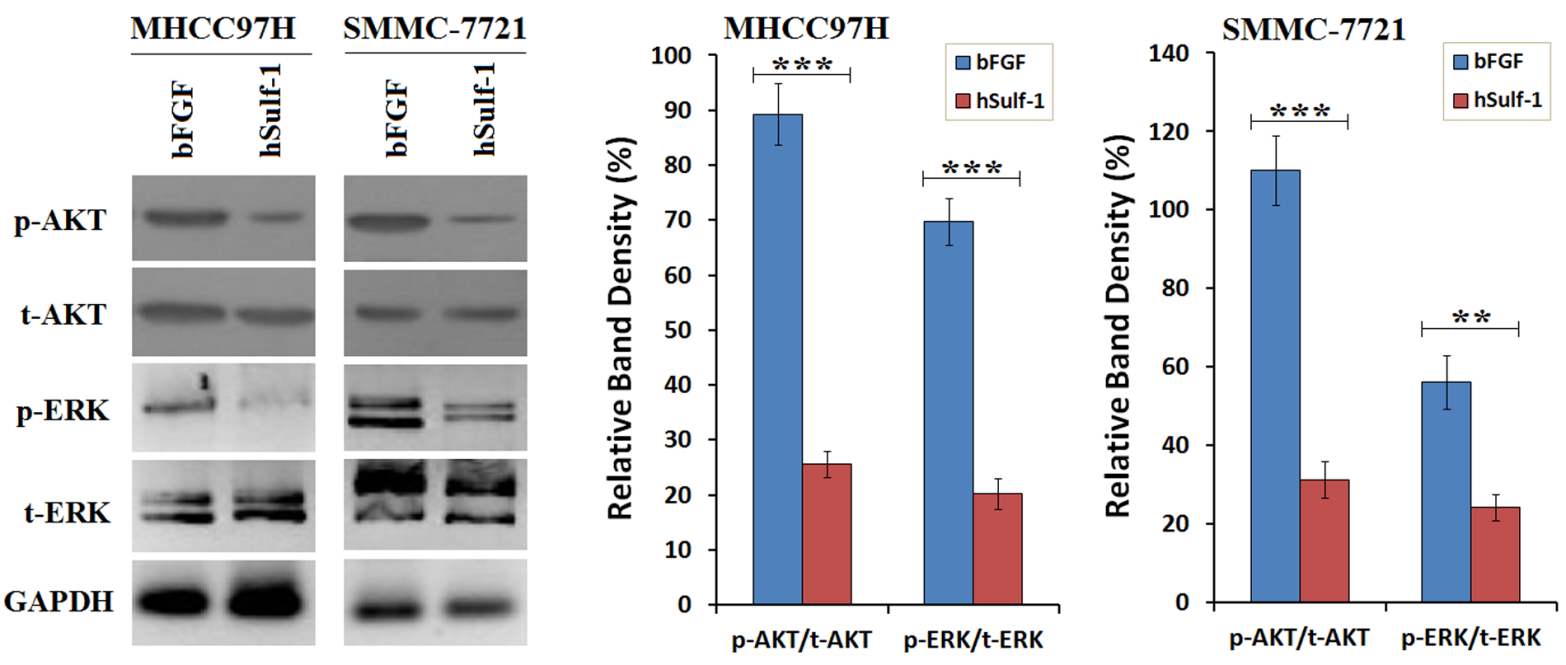
Suppression of bFGF-induced AKT and ERK signaling by hSulf-1 in HCC cells MHCC97H and SMMC-7721 cells were cultured in 6-well plates at 1×10^6^ cells/well with bFGF, and infected with adenovirus Ad5-hSulf1 at an MOI of 100 pfu/cell. Forty-eight h later, the expression of the indicated factors were examined by Western blot. GAPDH was used as the loading control. The densitometry analysis of p-AKT and p-ERK levels was performed, normalized with their corresponding total proteins (t-AKT and t-ERK, respectively). Columns, mean of three separate analyses; bars, standard deviation (SD); **P<0.01 and ***P<0.001.

As aforementioned, the expression of hSulf-1 can inhibit bFGF-stimulated cell cycle progression and induces cell apoptosis in HCC cells. The activity of downstream signaling was examined and found that bFGF increased the phosphorylation levels of AKT and ERK in the MHCC97H and SMMC-7721 cell lines. After infected with Ad5-hSulf1 and the HCC cells expressed hSulf-1, the phosphorylation levels of AKT and ERK were markedly decreased (Fig. 2).

### hSulf-1 downregulates Cyclin D1 and Survivin in HCC cells

**Figure 3:**
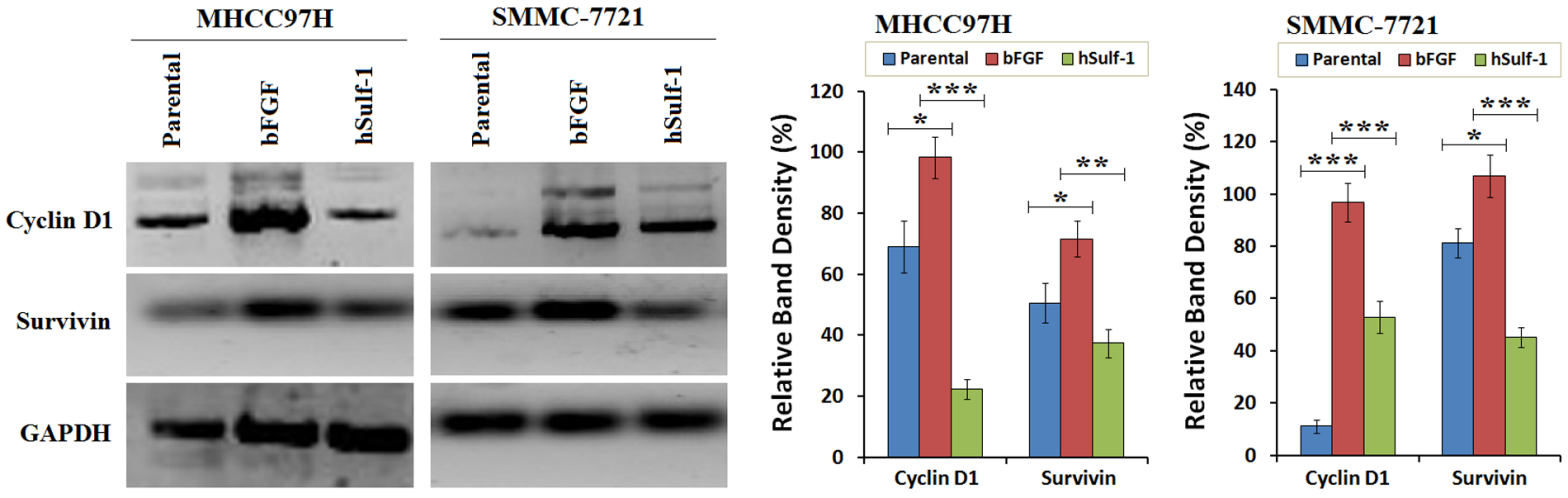
Downegulation of Cyclin D1 and Survivin by hSulf-1 in HCC cells MHCC97H and SMMC-7721 cells were cultured in 6-well plates at 1×10^6^ cells/well with or without bFGF, and infected with adenovirus Ad5-hSulf1 at an MOI of 100 pfu/cell. Forty-eight h later, the expression of the indicated factors were examined by Western blot. GAPDH was used as the loading control. The densitometry analysis of Cyclin D1 and Survivin levels was performed, normalized with GAPDH content. Columns, mean of three separate analyses; bars, standard deviation (SD); *P<0.05, **P<0.01 and ***P<0.001.

Cyclin D1 and Survivin were positively expressed in HCC cells. Under the condition of bFGF culture, the expression of Cyclin D1 and Survivin was particularly increased in the hSulf-1-negative MHCC97H and SMMC-7721 cell lines. After infected with Ad5-hSulf1, the expression of Cyclin D1 and Survivin was significantly decreased (Fig. 3). The results demonstrated that the effect of bFGF and hSulf-1 on cell cycle and apoptosis was mediated by the target factors of Cyclin D1 and Survivin.

### Expression of Cyclin D1 and Survivin was regulated by AKT and ERK signaling in HCC cells

**Figure 4:**
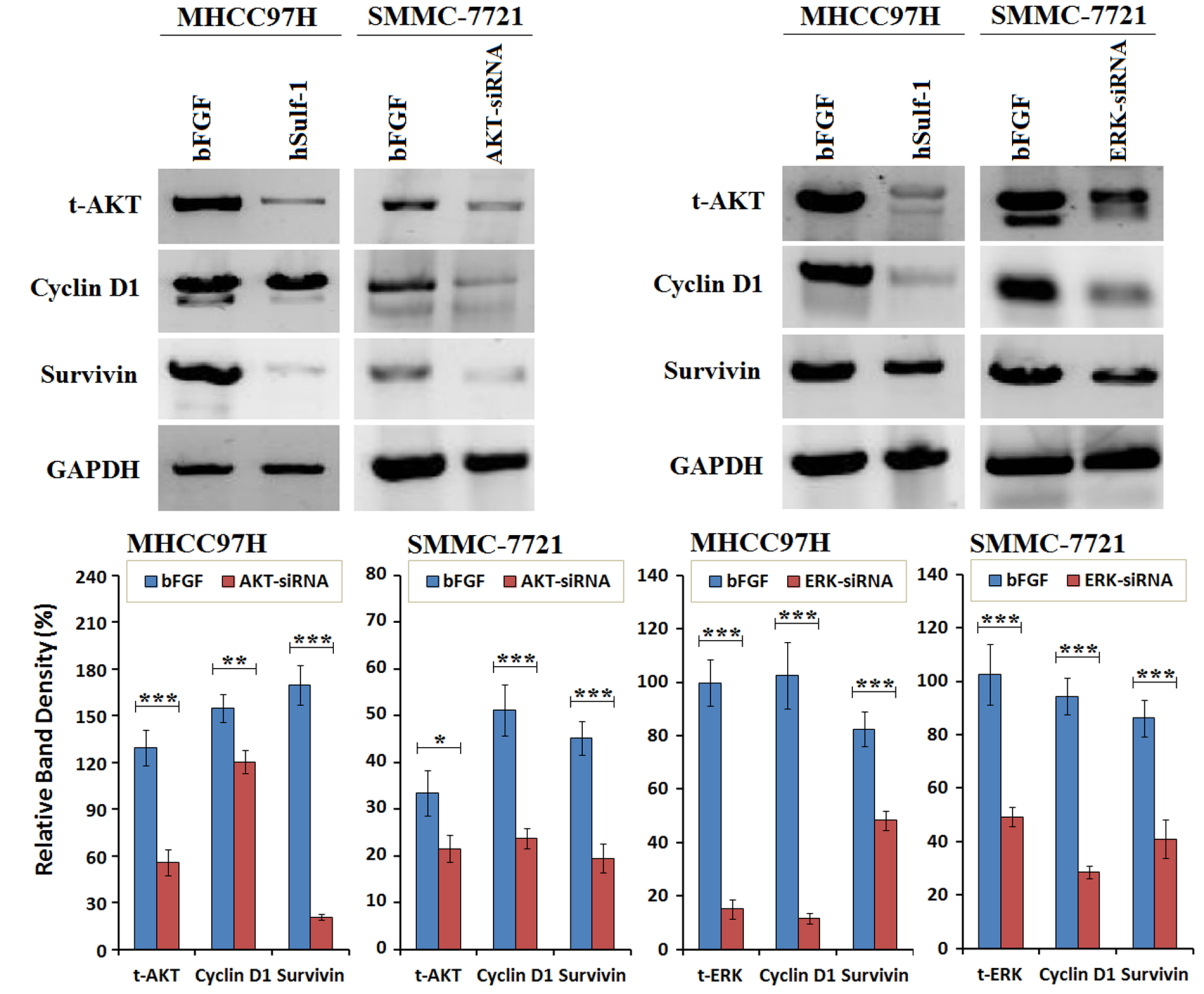
Cyclin D1 and Survivin was downregulated after silencing AKT and ERK expression in HCC cells MHCC97H and SMMC-7721 cells were cultured in 6-well plates at 1×10^6^ cells/well with bFGF, and transfected with AKT-siRNA or ERK-siRNA at a concentration of 10 μg/well. Cells were harvested at 48 h later and examined the expression of the indicated factors by Western blot. GAPDH was used as the loading control. The densitometry analysis of every factor was performed, normalized with GAPDH content. Columns, mean of three separate analyses; bars, standard deviation (SD); *P<0.05, **P<0.01 and ***P<0.001.

To confirm the superior-subordinate relationship of hSulf-1-mediated inhibition of Cyclin D1 and Survivin, the expression of AKT and ERK was silenced by their specific siRNAs. After transfected with AKT-siRNA, the expression of Cyclin D1 and Survivin was decreased in the MHCC97H and SMMC-7721 cell lines. After transfected with ERK-siRNA, the expression of Cyclin D1 and Survivin was also decreased in HCC cell lines. Cyclin D1 was markedly decreased after silencing AKT expression, and Survivin was markedly decreased after silencing ERK expression (Fig. 4). The results demonstrated that the expression of Cyclin D1 and Survivin was controlled under the AKT and ERK signaling pathways.

### hSulf-1 produces a synergic killing effect on HCC cells combined with rapamycin

**Figure 5:**
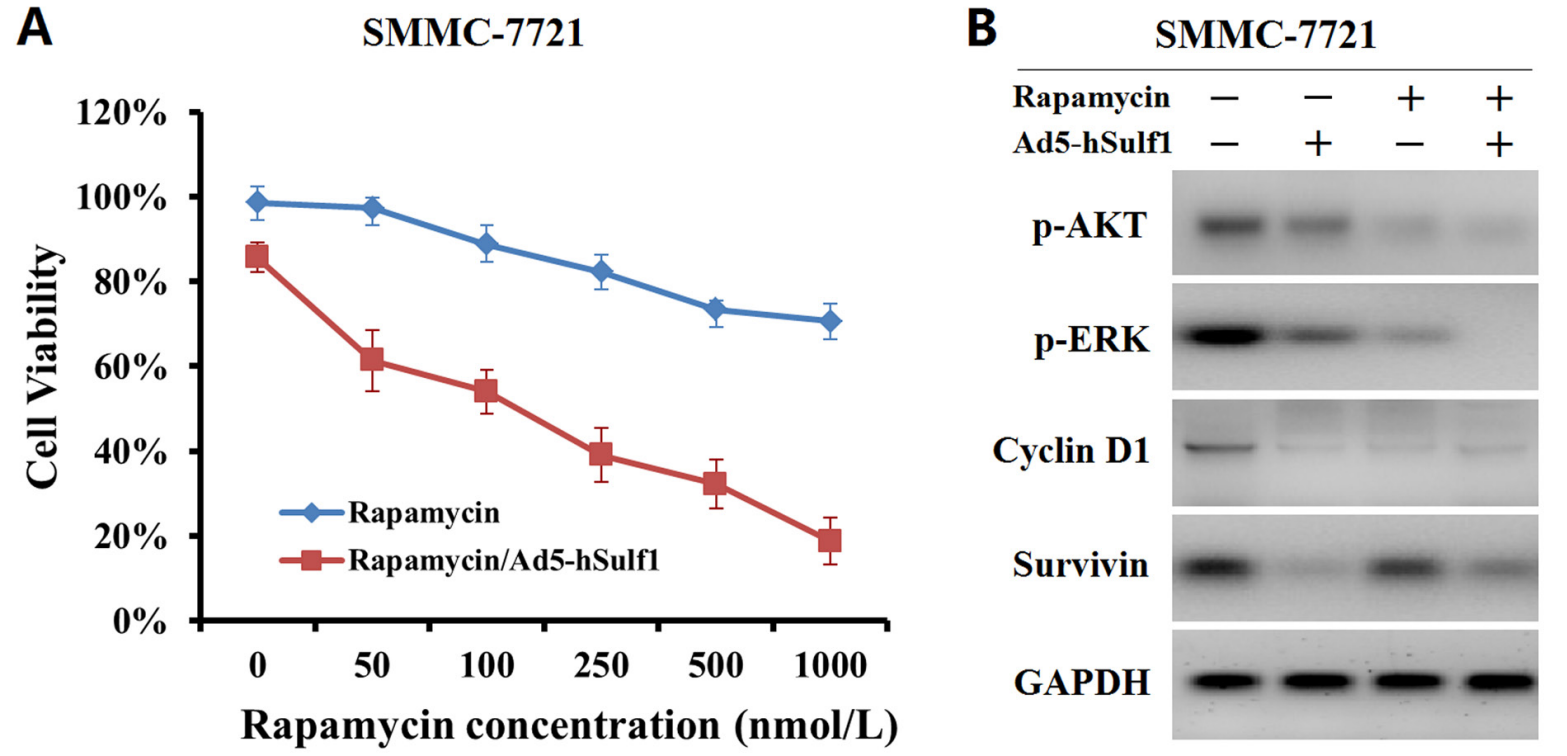
hSulf-1 produces a synergic inhibitory effect on HCC cell proliferation and signaling combined with rapamycin (A) SMMC-7721 cells were cultured in media containing 0 to 1,000 nmol/L rapamycin together with or without infection of adenovirus Ad5-hSulf1 at MOI of 100 pfu/cell. Cell viability was measured by Cell Proliferation Kit I after cultured for 48 h. (B) SMMC-7721 cells treated with 1,000 nmol/L rapamycin or/and 100 pfu/cell Ad5-hSulf1 were harvested and detected for the expression of AKT, ERK, Cyclin D1 and Survivin by Western blot. GAPDH was used as the loading control.

To examine the effect of hSulf-1 combined with the mTOR inhibitor, rapamycin, SMMC-7721 cell viability was measured after treated with Ad5-hSulf1 and rapamycin. With increase of rapamycin concentration, cell viability was gradually decreased in SMMC-7721 cells, being (70.62 ± 4.16) % at concentration of 1,000 nmol/L. Cell viability was sharply declined in Ad5-hSulf1-preconditioned SMMC-7721 cells, being (18.69 ± 5.55) % at rapamycin concentration of 1,000 nmol/L (Fig. 5A). The parental and preconditioned SMMC-7721 cells were detected for the expression of AKT, ERK, Cyclin D1 and Survivin. The results showed that Ad5-hSulf1 at MOI of 100 pfu/cell led to a weaker decrease of p-AKT and p-ERK expression, but a stronger decrease of Cyclin D1 and Survivin expression, compared with rapamycin at concentration of 1,000 nmol/L (Fig. 5B).

### Adenovirus-mediated hSulf-1 expression exerts antitumor efficacy by downregulating Cyclin D1 and Survivin in HCC xenografts in nude mice

**Figure 6:**
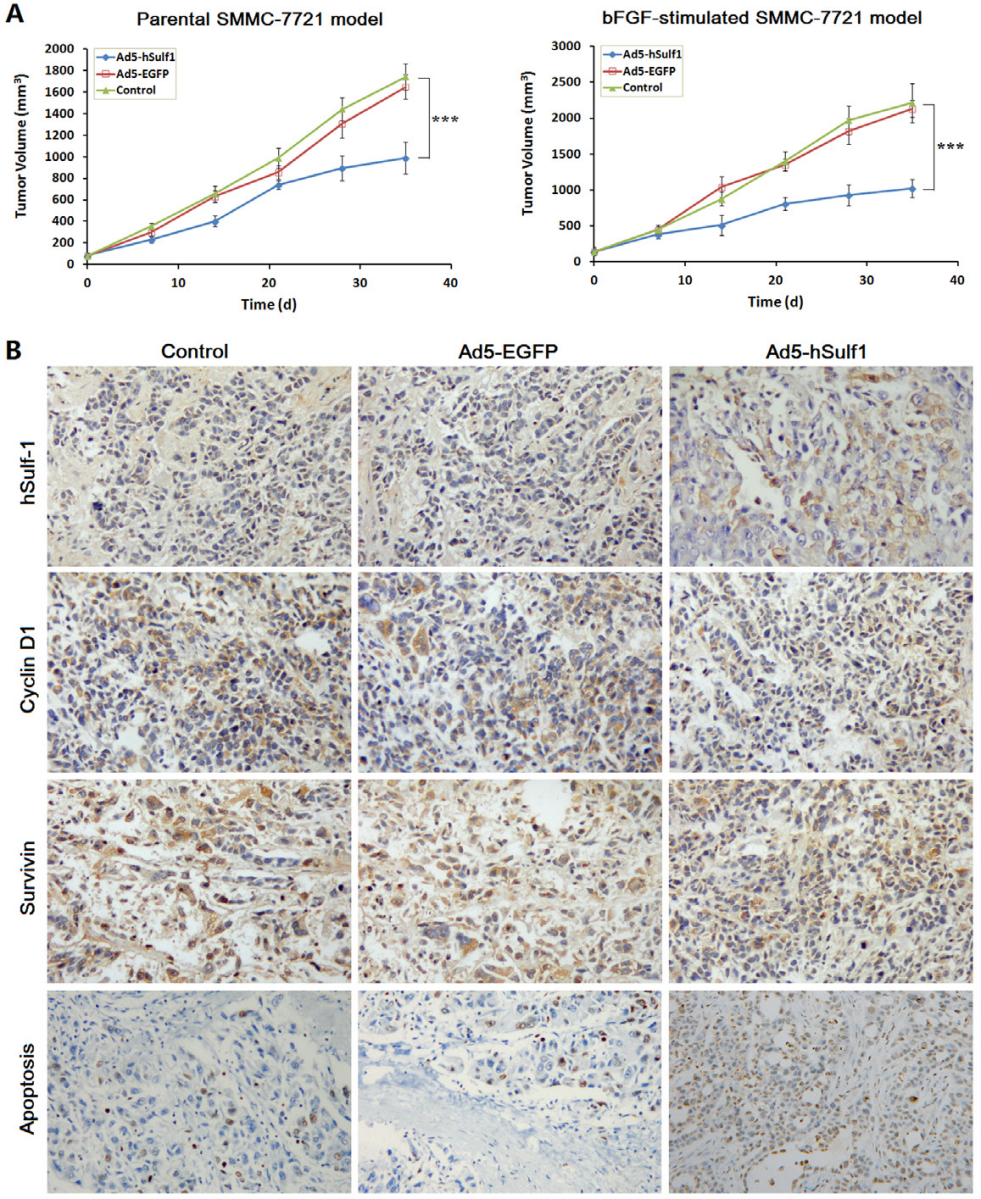
hSulf-1-mediated antitumor efficacy in HCC xenografts in nude mice (A) The xenograft tumors in nude mice were established by subcutaneously injection of the parental SMMC-7721 cells and the bFGF-cultured SMMC-7721 cells, respectively, 10^6^ cells per mouse. Every model was separated 3 groups, 5 mice per group. After treated with adenoviruses Ad5-hSulf1 and Ad5-EGFP, tumor volume was measured according to the previously reported method and formula [13,39]. Lines, mean of tumor volume (n=5); bars, standard deviation (SD); ***P<0.001. (B) Immunohistochemical examination of the indicated factor expression in the xenograft tumors, original magnification ×200. The data of all the quantitative analyses in every group were showed in Table 2.

The xenograft tumor models were established with both the parental SMMC-7721 cells and the bFGF-cultured SMMC-7721 cells. Apparently, the bFGF-cultured SMMC-7721 model had characteristics of earlier tumor formation and quicker growth than the parental SMMC-7721 model. After treated with Ad5-hSulf1, the tumor inhibitory rates were 43.43% and 53.92% in the parental SMMC-7721 model and the bFGF-cultured SMMC-7721 model, respectively, demonstrating that the tumor growth suppression of Ad5-hSulf1 was more effective in the bFGF-cultured SMMC-7721 model (Fig. 6A). There was no difference between the Ad5-EGFP-treated group and the blank control group.

The xenograft tumors of the bFGF-cultured SMMC-7721 model were examined immunohistochemically. The expression of hSulf-1 and the percentage of apoptosis were increased. The expression of Cyclin D1 and Survivin was decreased in the Ad5-hSulf1-treated group, compared with the blank control group (Fig. 6B; Table 2).

**Table 2:** Effect of hSulf-1 on the regulators of cell cycle and apoptosis in HCC xenograft tumors

Animal Group	Positive Cells (% of total cells counted)			
	hSulf-1	Cyclin D1	Survivin	Apoptosis
Control	0.56±0.08	41.21±9.54	83.12±12.33	5.48±0.96
Ad5-EGFP	0.32±0.06	37.37±7.63	78.58±10.26	7.79±1.21*
Ad5-hSulf1	54.66±8.42***	11.52±5.49**	22.92±4.15***	58.57±11.22***

Notes: SNK-q test, **P<0.01, ***P<0.001 compared with the control group.

## DISCUSSION

The hSulf-1 expression can diminish the cascade phosphorylation of a series of kinases including the extracellular signal-regulated kinase (ERK) and serine/threonine kinase (AKT), and followed by inactivation of downstream signaling pathways. This mechanism plays a distinct part in the inhibition of malignant transformation and cancer growth [20-24]. Our previous study showed that hSulf-1 is inactivated in majority of human cancers including hepatocellular, breast, gastric, renal and colon cancers, compared with their adjacent normal tissues, and reactivation of hSulf-1 can suppress cancer cell proliferation and xenograft tumor growth by inhibiting the activity of ERK and AKT signaling pathways [11,12].

The bFGF expression can promote cancer cell proliferation by activating the PI3K/AKT and MAPK/ERK pathways in cancer [14,15]. bFGF binds its receptor on cell surface and activates the downstream signaling pathways, then functions the mitogenic activity and promotes cancer cell cycle progression [16-19]. Since hSulf-1 can desulfate cell surface HSPGs and block the bFGF-mediated receptor tyrosine kinase signaling [25,26], there is a strong reason to suppose that hSulf-1 can regulate cancer cell cycle and apoptosis. However, this function and its molecular mechanism have been rarely reported and not been well known yet.

The current study found that the bFGF product promotes cell cycle progression and inhibits cell apoptosis in hSulf-1-negative HCC cell lines and HCC xenograft tumors, the outstanding feature was that the percentages of G0/G1-phase and the percentages of cell apoptosis were decreased and the percentages of S-phase were increased in the HCC cell lines, but the changes in cell cycle and apoptosis had no significant difference in the normal hepatocyte cell lines. By reactivation of hSulf-1 expression and function in hSulf-1-negative HCC cell lines, the effect of bFGF on cell cycle progression and apoptosis was reversed, demonstrating that there is a relationship between hSulf-1 and bFGF functions.

To further investigate the molecular mechanism of hSulf-1 effect on cancer cell cycle and apoptosis, we found that the reactivation of hSulf-1 expression and function in hSulf-1-negative HCC cell lines can downregulate the phosphorylation of AKT and ERK and inhibit the activity of bFGF-induced AKT and ERK signaling in HCC cells, then finally led to cell cycle arrest and cell apoptosis by suppressing the expression of Cyclin D1. This phenomenon was approved in HCC xenograft tumor in nude mice. Cyclin D1 is the key and initial regulator in cell cycle control [27-29]. By binding to CDK4/6, Cyclin D1 activates CDK4/6 and promotes the phosphorylation of Rb protein, which makes a release of E2F-1 transcription factor from Rb-E2F-1 complex. The released E2F-1 activates the transcription of many important cell cycle regulatory genes to accelerate cell cycle progression [30,31]. Silencing Cyclin D1 expression by miR-17/20 can induce G1/S cell cycle arrest and inhibit cancer cell proliferation [32]. Cyclin D1 is considered as an oncogene because of its overexpression with high activity in most human cancers [33,34].

Moreover, the study also demonstrated that inhibition of AKT and ERK signaling by hSulf-1 led to a decrease of Survivin expression in HCC cell lines and xenograft tumors. The overexpression of Cyclin D1 also increases Survivin expression [35]. Because Survivin plays an important role in maintaining cancer cell survival, therefore, Survivin is a crucial target for cancer therapy [36]. The activity of AKT/mTOR signaling is frequently shown to be aberrantly up-regulated in HCC. The allosteric AKT inhibitor, MK-2206, and γ-tocotrienol can down-regulate AKT/mTOR signaling activity, then induce cell cycle arrest in the G0/G1 phase of the cell cycle, cause apoptosis and reduce tumor growth [37,38]. There was evidence that the inhibition of AKT signaling can reduce Survivin expression in HCC cancer cells [39]. Survivin belongs to the family of apoptosis inhibitors (IAPs) and is highly expressed in majority of cancers, which makes it an attractive target for cancer intervention and therapy [40]. Given that the hSulf-1-mediated decrease of Cyclin D1 and Survivin in HCC cancer cells, it is concluded that the molecular mechanism by which the hSulf-1 expression reverses the bFGF-mediated cell cycle progression and cell apoptosis inhibition may be through the modification of AKT- and ERK-regulated Cyclin D1 and Survivin signaling pathways. Since AKT/mTOR appears to play a central role in signaling caused by many growth factors to regulate cell proliferation and survival, some mTOR inhibitors, such as rapamycin and CCI-779, are tested as anti-cancer agents [37,41,42]. We also examined the killing effect of rapamycin with or without hSulf1 expression in SMMC-7721 cells, and the expression of p-AKT, p-ERK, Cyclin D1 and Survivin, and further demonstrated that hSulf-1 enhances the inhibitory effect of rapamycin on cell survival signaling and produces a synergic killing effect on HCC cells combined with rapamycin.

To conclude, this study gave a convincing evidence to demonstrate that hSulf-1 is a sulfatase and can reverse the effect of bFGF on cell cycle and cell apoptosis by inhibiting bFGF-mediated receptor tyrosine kinase signaling and decreasing the expression of Cyclin D1 and Survivin. Therefore, the current study suggested that hSulf-1 plays an important role in the regulation of cell cycle and apoptosis and may be considered as a potential target in HCC therapy.

## MATERIALS AND METHODS

### Cell lines and cell preparation

The HCC cell lines, MHCC97H, MHCC97L, SMMC-7721, BEL-7404, and the normal hepatocyte lines, WRL-68 and L02, were purchased from the Shanghai Cell Bank (Shanghai Institute for Biological Science, Chinese Academy of Science, Shanghai, China). All cell lines were cultured in media according to the provider's instruction. The preconditioned cells used in the in vitro experiments were cultured in media containing 20 ng/ml bFGF (Sigma-Aldrich, Shanghai, China), or in media containing 0 to 1,000 nmol/L rapamycin (Sigma-Aldrich, Shanghai, China). Cells were also preconditioned by infection of adenovirus (Ad5-hSulf1, Ad5-EGFP) [13] at a multiplicity of infection (MOI) of 100 pfu/cell. All the parental and preconditioned cells were cultured for 48 h, and harvested.

### Analysis of cell cycle and apoptosis by flow cytometry (FCM)

Cells were cultured in 6-well plates at 1×10^6^ cells/well and harvested. The procedures of propidium iodide (PI) staining, Annexin V/PI staining, and the examinations of cell cycle and apoptosis by flow cytometer (FACS420, BD Biosciences, San Jose, CA) were operated as described previously [43].

### Examination of gene expression by Western blot

The expression of indicated factors was examined by Western blot, with the primary antibodies including rabbit anti-hSulf-1, mouse anti-human total AKT (t-AKT), rabbit anti-human phosphorylated AKT (p-AKT) (Santa Cruz Biotechnology, Santa Cruz, CA,USA); rabbit anti-human total ERK1/2 (t-ERK) and phosphorylated ERK (p-ERK), mouse anti-human Cyclin D1 (Cell Signaling Technology, Danvers, MA, USA); rabbit anti-human Survivin (RD Systems Inc, Minneapolis, MN,USA); and mouse anti-human glyceraldehydes 3-phosphate dehydrogenase (GAPDH) (Kangchen Bio-tech, Shanghai, China). Signals were visualized by ECL chemiluminescence. Equal protein loading was assessed by the expression of GAPDH.

### Silence of AKT and ERK expression by siRNAs

The siRNAs of AKT and ERK were synthesized (Biotechnology Co., Wuhan, China). The Akt-siRNA targets the sequence 1338-1360 bp (21 bp target + 2 bp overhang: gactacctgcactcggagaagaa) of Akt1 gene (NM_005163.2). The ERK-siRNA targets the sequence 903-925 bp (acctgaattgtatcatcaacat) of ERK1 gene (NM_002746.2). A mock control siRNA (Ctrl-siRNA, gacttcataaggcgcatgctgtc) was synchronously synthesized. Totally 10^6^ cells were seeded in 6-well plates and transfected with siRNAs at a concentration of 10 μg/well using Lipofectamine 2000 (Invitrogen, Casbad, CA). Cells were harvested at 48 h later after transfection and examined for the indicated factors.

### Cell viability experiment

The parental and preconditioned cells were cultured in 96-well plates at 1×10^4^ cells/well for 48 h, and measured by Cell Proliferation Kit I (Roche Diagnostics GmbH, Germany) to determine the cell viability according to the kit protocol.

### Animal models and *in vivo* experiments

The animal study was approved by the animal ethics committee of Second Military Medical University (Shanghai, China). All animal study procedures were performed in compliance with the Guide for the Care and Use of Laboratory Animals of the US Department of Health and Human Services.

The xenograft tumor models were established by subcutaneously injecting the parental SMMC-7721 cells and the bFGF-cultured SMMC-7721 cells (10^6^ cells per mouse), respectively, into the right flanks of 30 BALB/c (nu/nu) mice (Shanghai Experimental Animal Center, Chinese Academy of Sciences, Shanghai, China). The xenograft tumors were about 0.5 cm in diameters after two weeks later in the bFGF-cultured SMMC-7721 model and after three weeks later in the parental SMMC-7721 model. Mice in every model were separated randomly into 3 groups: the Ad5-hSulf1-treated group, Ad5-EGFP-treated group and control groups, 5 mice per group. The Ad5-hSulf1 and Ad5-EGFP groups were intratumorally injected with 2×10^8^ pfu viruses, one injection every other day and totally 5 injections with total dose of 10^9^ pfu viruses per mouse. Mice in the control group were injected with the same volume of viral preservation solution (10 mmol/L Tris-HCl pH 8.0, 4% sucrose, 2 mmol/L MgCl_2_). Tumor volume was measured according to the previously reported method and formula [13,39]. The observation was stopped 35 days later after treatments, when the tumor volume in any group was over the standard (2000 mm^3^) approved by the animal ethics committee.

All mice were painlessly killed, and the tumors were collected from the bFGF-cultured SMMC-7721 model. The expression of hSulf-1, Cyclin D1 and Survivin was examined by immunohistochemistry, and cell apoptosis was detected by the TdT-mediated dUTP nick end labeling (TUNEL) according to reference [44]. The percentages of positive cells were counted in each section under 5 high-power fields (objective lens: 40×).

### Statistical analysis

The experimental data from 3 times of independent in vitro experiments, as well as in vivo experimental data from 5 mice per group, were presented as ‘mean ± standard deviation (SD)’, and analyzed by one-way analysis of variance (ANOVA). When the data were statistically different among the multiple groups, the SNK-q test was used to conduct the multiple comparison. The statistical analysis software package PASW Statistics 18 was used. *P* values less than 0.05 were considered statistically significant.
